# Expression Profiling of Plant Cell Wall-Degrading Enzyme Genes in *Eucryptorrhynchus scrobiculatus* Midgut

**DOI:** 10.3389/fphys.2020.01111

**Published:** 2020-09-04

**Authors:** Peng Gao, Zhenkai Liu, Junbao Wen

**Affiliations:** ^1^Beijing Key Laboratory for Forest Pest Control, Beijing Forestry University, Beijing, China; ^2^Research Institute of Forestry New Technology, Chinese Academy of Forestry, Beijing, China

**Keywords:** carbohydrate esterase, cellulase, glycoside hydrolase, hemicellulase, pectinase, polysaccharide lyase, transcriptome

## Abstract

In China, the wood-boring weevil *Eucryptorrhynchus scrobiculatus* damages and eventually kills the tree of heaven *Ailanthus altissima*. To feed and digest the cell wall of *A. altissima*, *E. scrobiculatus* requires plant cell wall-degrading enzymes (PCWDEs). In the present study, we used next-generation sequencing to analyze the midgut transcriptome of *E. scrobiculatus.* Using three midgut transcriptomes, we assembled 21,491 unigenes from 167,714,100 clean reads. We identified 25 putative PCWDEs, including 11 cellulases and 14 pectinases. We constructed phylogenetic trees with a maximum likelihood algorithm to elucidate the relationships between sequences of the PCWDE protein families and speculate the functions of the PCWDE genes in *E. scrobiculatus*. The expression patterns of 17 enzymes in the midgut transcriptome were analyzed in various tissues by quantitative real-time PCR (RT-qPCR). The relative expression levels of 12 genes in the midgut and two genes in the proboscis were significantly higher than those in the other tissues. The proboscis and midgut are the digestive organs of insects, and the high expression level indirectly indicates that these genes are related to digestion. The present study has enabled us to understand the types and numbers of the PCWDEs of *E. scrobiculatus* and will be helpful for research regarding other weevils’ PCWDEs in the future.

## Introduction

Plant cell walls are the most abundant and useful source of biomass on Earth. Coleoptera insects can spread into most habitats worldwide and have become the most species-rich insect order on Earth owing to their mostly herbivorous nature (digesting plant cell walls as nutrients) (Pauchet et al., 2010; Schuman and Baldwin, 2016; Antony et al., 2017). Herbivorous insects are thought to rely on gut microorganisms to digest plant cell walls until the endogenous cellulase genes from termites are identified (Watanabe et al., 1998; Calderón-Cortés et al., 2012). These endogenous insect genes that degrade plant cell walls are called plant cell wall degrading enzymes (PCWDEs). Studies have shown that herbivorous insects obtain a variety of PCWDE families through horizontal gene transfer (HGT) and then use plant cell walls more efficiently through gene replication and functional diversification (Calderón-Cortés et al., 2012; Kirsch et al., 2014, 2016; Busch et al., 2019). Since endogenous PCWDEs are thought to play important roles in the evolution of plant–insect interactions and insect diversification (Calderón-Cortés et al., 2012), a large number of reports on endogenous PCWDEs in herbivorous insects have been published in recent years (Watanabe and Tokuda, 2010; Pauchet et al., 2014; Kirsch et al., 2016; Antony et al., 2017). The advent of next-generation sequencing methods has enabled the identification of a growing number of endogenous PCWDEs in beetles, such as *Rhynchophorus ferrugineus*, *Apriona japonica*, and *Phaedon cochleariae* (Roy et al., 2012; Pauchet et al., 2014; Antony et al., 2017).

The degradation of plant cell walls by phytophagous insects is the primary requirement for utilizing host plants (Antony et al., 2017). Plant cell wall polysaccharides mainly consist of cellulose as well as hemicellulose and pectin (Cosgrove, 2005), and PCWDEs include cellulase, hemicellulase, and pectinase (Juge, 2006). Different hydrolase families of PCWDEs have been identified in many insect species (Watanabe et al., 1998; Calderón-Cortés et al., 2012), and these digestive enzymes play key roles in insect feeding diversity (Pauchet et al., 2010; Watanabe and Tokuda, 2010; Antony et al., 2017). Previous reports indicated that insect PCWDEs include three carbohydrate-active enzymes (CAZymes), namely glycoside hydrolase (GH), carbohydrate esterase (CE), and polysaccharide lyase (PL) (Cantarel et al., 2009; Pauchet et al., 2010; Watanabe and Tokuda, 2010; Calderón-Cortés et al., 2012). Endogenous insect cellulases include GH5, GH9, GH45, and GH48 (Pauchet et al., 2010; Watanabe and Tokuda, 2010; Calderón-Cortés et al., 2012). In addition to the GH48 family, cellulase activity has been detected in other families (Calderón-Cortés et al., 2012; Kirsch et al., 2014, 2016; Busch et al., 2018, 2019). However, only GH48 in insects is confirmed to have chitinase rather than cellulase activity (Fujita et al., 2006). The most common hemicellulases are xylanase and xyloglucanase (Calderón-Cortés et al., 2012). Only two endogenous genes encoding xylanases were found in *Phaedon cochleariae*, and functions have been verified (Girard and Jouanin, 1999; Calderón-Cortés et al., 2012; Pauchet and Heckel, 2013). Studies have shown that insects use the pectinases GH28 (polygalacturonase), CE8 (pectin methylesterase), and PL4 (rhamnogalacturonan lyase) to degrade the cell wall pectin network (Pauchet et al., 2010; Calderón-Cortés et al., 2012; Kirsch et al., 2016). The GH28 gene is ubiquitous in insects including the Curculionoidea. Weevils have both CE8 and GH28 genes (Pauchet et al., 2010; Calderón-Cortés et al., 2012; Kirsch et al., 2016). The functions of many CE8 and GH28 genes have been verified (Kirsch et al., 2014, 2016). The GH28 genes exhibit other enzyme activities in addition to pectinase activity, and some GH28 genes for which enzyme activities have not been detected may be pseudogenes (Kirsch et al., 2014). The PL4 genes have only been identified in weevils, such as *Dendroctonus ponderosae* and *Hypothenemus hampei*, and the function of these genes has not been identified (Pauchet et al., 2010; Keeling et al., 2013; Vega et al., 2015).

The weevil *Eucryptorrhynchus scrobiculatus* Motschulsky (Coleoptera: Curculionidae) is an important pest of the tree of heaven (*Ailanthus altissima* Swingle). It has an extensive geographical distribution across China (Yu et al., 2013; Liu and Wen, 2016; Yang et al., 2017) and can, therefore, cause serious damage (Ji et al., 2017a). The entire *E. scrobiculatus* life cycle occurs on the tree of heaven. The larvae and adults feed on the plant tissues and sap, generally feeding on weak and mechanically injured trees rather than healthy ones. As such, it is a secondary pest of *A. altissima* (McAvoy et al., 2013). *E. scrobiculatus* adults attack the trees while the larvae simultaneously attack the roots. If unchecked, this weevil may eventually kill its host (Ding et al., 2006). The adult insect is very harmful to *A. altissima* (Ji et al., 2017b), so the cellulase activity of the gut extract was tested (Chen, 2016). Chen’s study shows that the cellulase activity of the male *E. scrobiculatus* gut extract is significantly higher than that of female *E. scrobiculatus* gut extract (Chen, 2016). In the present study, we identified PCWDEs in *E. scrobiculatus* midgut by high-throughput sequencing and investigated their expression patterns. PCWDEs that have previously been functionally verified in insects were used to predict their function in *E. scrobiculatus*. Given the intrinsic nature of the wood boring weevil, our results are fundamental for studying the molecular mechanism of the digestion of plant cell walls in *E. scrobiculatus*. PCWDEs have the potential for pest management and biofuels.

## Materials and Methods

### Insect Rearing and Tissue Collection

Adult *E. scrobiculatus* were collected in Wutongshu Town, Pingluo County, Ningxia Hui Autonomous Region, China (38°16′86″N, 106°29′71″E) in June 2017. The insects were reared in a breathable plastic box (50 × 40 × 40 cm) containing perennial *A. altissima* branches. The plastic box was delivered to the Beijing Key Laboratory for Forest Pest Control in Beijing, China. There, the insects were frozen in liquid nitrogen, surface-sterilized with 75% (v/v) alcohol, and rinsed with sterile water. Dissection was performed by using an anatomical mirror (Leica, SE6) on ice on a clean bench. Various tissues (Head, leg, proboscis, foregut, midgut, and hindgut) were stored separately at -80°C. The midgut (the intestinal section between the cardiac valve and the base of the Malpighian tubule) was excised for RNA extraction.

### Total RNA Extraction

Five midguts were used per RNA extraction. These were performed as three independent biological replicates with a RNeasy Plus kit (Qiagen, Hilden, Germany) following the manufacturer’s instructions. The total RNA was treated with DNase. RNA was quantitated with a Bioanalyzer 2100 (Agilent Technologies, Santa Clara, CA, United States) and monitored with a NanoDrop 8000 (Thermo Fisher Scientific, Waltham, MA, United States). Only high-quality RNA samples were used to construct the sequencing library, with optical density OD_260__/__/__280_ = 1.8 − 2.2, OD_260__/__230_ ≥ 2.0, RIN ≥ 6.5 and, 28S ribosomal RNA:18S ribosomal RNA ≥ 1.0. The RIN indicates the integrity of the RNA sample and is in the range of 1–10. RNA integrity increases with RIN.

### Library Construction and Illumina Sequencing

Three RNA-seq transcriptome libraries were prepared using a TruSeq^TM^ RNA sample preparation kit from Illumina (San Diego, CA, United States) and 5 μg RNA. Messenger RNA was isolated with oligo (dT) beads by the polyA selection method and fragmented in fragmentation buffer. Double-stranded cDNA was synthesized with a SuperScript double-stranded cDNA synthesis kit (Invitrogen, Carlsbad, CA, United States) and random hexamer primers (Illumina, San Diego, CA, United States). The cDNA was subjected to end-repair, phosphorylation, and ‘A’ base addition according to the Illumina library construction protocol. The libraries were assembled by selecting 200 − 300 bp cDNA fragments on a 2% low-range ultra-agarose gel, followed by PCR amplification with Phusion DNA polymerase (New England Biolabs (NEB), Ipswich, MA, United States) for 15 cycles. After quantitation with a TBS380 fluorometer (Turner BioSystems, Sunnyvale, CA, United States), the paired-end RNA-seq library was sequenced with an Illumina HiSeq 4000 (Illumina, San Diego, CA, United States). Read lengths of 150 bp and 6.6 Gb were targeted for sequencing. There were three biological replicates per experiment.

### Assembly and Functional Annotation

Raw paired-end reads were trimmed and subjected to quality control with SeqPrep^1^ and Sickle^2^ using their default parameters. Clean reads were assembled with Trinity^3^. A 200-bp minimum contig length and k = 25 were used for the assembly. Normalization was performed *in silico*. The largest alternative splicing variant in the Trinity output for each unigene was selected as a unigene. The unigenes were searched against the NCBI non-redundant (NR) protein database using an e-value cutoff of 10^–5^. The open reading frame (ORF) of each unigene was determined with the NCBI ORF Finder tool^4^. The BLAST2GO program was used to obtain GO annotations for the unigenes and identify putative biological processes, molecular functions, and cellular components. Transcript expression levels were calculated by the fragments per kilobase of exon per million mapped reads (FRKM) method (Li and Dewey, 2011). The final FPKM is the average FPKM of three biological replicates.

### Identification of Endogenous PCWDEs

All candidate GH9, GH28, GH45, GH48, PL4, and CE8 enzymes were manually checked with the NCBI BLASTx tool (Altschul et al., 1990). Unigenes potentially encoding the PCWDEs were confirmed and categorized into glycoside hydrolase (GH) families with HmmSearch (Zhang et al., 2018). If genes have incomplete open reading frames in the transcriptome, the complete open reading frames were obtained with a SMARTer RACE 5’/3’ Kit (TaKaRa Bio Inc., Kusatsu, Shiga, Japan) according to the manufacturer’s instructions.

### Phylogenetic Analysis of *E. scrobiculatus* PCWDEs

The sequence homology and evolutionarily conserved features of *Eucryptorrhynchus scrobiculatus* PCWDEs were analyzed by reconstructing phylogenetic trees with *E. scrobiculatus* protein sequences and homologous sequences from beetles and other insects. Closely related sequences from other insects were obtained from National Center for Biotechnology Information (NCBI) for each gene subfamily. A reference sequence whose structure was already elucidated was retrieved from the CAZy database. Multiple sequence alignment was performed with ClustalW (Price et al., 2010). Phylogenetic trees were constructed by the maximum-likelihood method in IQ-TREE (Minh et al., 2020). Phylogenetic trees were color-coded and arranged using FigTree v1.4.4 and TBtools v1.046 (Chen et al., 2020).

### Tissue Expression Analysis of *E. scrobiculatus* PCWDEs

The expression profiles of PCWDE genes that were identified in the midgut transcriptome and whose FPKM was greater than 10 in various tissues (head, leg, proboscis, foregut, midgut, and hindgut) were analyzed by RT-PCR and RT-qPCR. Simultaneously, the only GH9 gene was also subjected to these analyses. The total RNA was processed as previously described. The first-strand cDNA was synthesized from the total RNA with a PrimeScript RT reagent kit and gDNA Eraser (TaKaRa Bio Inc., Kusatsu, Shiga, Japan) according to the manufacturer’s instructions. Two hundred nanograms cDNA was used for the RT-PCR and RT-qPCR templates. The RT-PCR specific primers were designed with PRIMER3 (Untergasser et al., 2012) using the following parameters: melting temperature (T_*m*_) = 60°C; GC content = 40% − 60%; and product size = 100 − 200 bp. The primer sequences are listed in Supplementary Table S3. The PCR was performed using PrimeSTAR Max DNA Polymerase (TaKaRa Bio Inc., Kusatsu, Shiga, Japan) with an initial denaturation step at 98°C for 1 min followed by 35 cycles at 98°C for 20 s, 60°C for 15 s, 72°C for 1 min, and a final extension of 5 min at 72°C. The PCR products were electrophoretically separated on 2% agarose gels and verified by DNA sequencing.

The RT-qPCR analysis was conducted with an Applied Biosystems StepOnePlus^TM^ (Thermo Fisher Scientific, Waltham, MA, United States) in 20-μL reaction volumes consisting of 10 μL SYBR Premix Ex Taq II, 0.8 μL of each primer (10 mM), 2 μL sample cDNA (2.5 ng RNA), 0.4 μL ROX, and 6 μL sterile distilled water. Thermal cycling was performed at 95°C for 30 s (pre-cycling) followed by 40 cycles at 95°C for 10 s, 57°C for 10 s, 60°C for 20 s, 95°C for 10 s, and a melting curve analysis at 65°C for 5 s. The annealing temperature was increased by 0.5°C per cycle to 95°C. All reactions were performed in triplicate. Gene expression levels in the adult gut tissues were compared with those in the other tissues and quantitated by the comparative 2^–ΔΔ*Ct*^ method (Livak and Schmittgen, 2001) with Applied Biosystems StepOnePlus^TM^ software (Applied Biosystems, Foster City, CA, United States). The *E. scrobiculatus RPL13* gene was the endogenous control for normalizing PCWDE expression. The PCR amplifications was performed separately for each tissue using the same primers as those for RT-PCR amplification (Supplementary Table S1).

### Statistical Analysis

ONE-way analysis of variance (ANOVA) with Tukey’s honestly significant difference (HSD) test was used to determine the differences in the expression of each PCWDE in the six tested tissues (head, leg, proboscis, foregut, midgut, and hindgut). The statistical analysis was performed using SPSS (version 20.0) and significant expression was considered for *P* < 0.05 (IBM Corp, Armonk, NY, United States). Cycle threshold (Ct) values are presented as mean ± SE.

## Results

### Transcriptome Sequencing and Unigene Assembly

The three midgut cDNA libraries (ESM1, ESM2, and ESM3) for *E. scrobiculatus* were sequenced in an Illumina HiSeq 4000 system (Illumina, San Diego, CA, United States). The raw reads and the Q20 and Q30 base call accuracies of the three ESM samples are shown in Supplementary Table S1. The adaptors were trimmed, low-quality sequences were removed, and the ESM1, ESM2, and ESM3 sequences were blended, spliced, and assembled. We obtained 21,491 unigenes with N_50_ = 2,736 bp and mean length = 1,520 bp (Supplementary Table S1). The assembled unigene lengths ranged from 201–29,088 bp (Supplementary Figure S1). The raw *E. scrobiculatus* reads were deposited in the NCBI SRA database under GenBank accession number SRP070604.

### Homology Searching and Gene Functional Annotation

Unigenes were used as queries for BLASTx and BLASTp searches against protein databases. The E-value cutoff was 10^–5^. There were 4,269 (19.86%), 10,041 (46.72%), 6,127 (28.51%), 8,482 (39.47%), 3,540 (16.47%), 5,491(25.55%), 361 (1.68%), and 9,197 (42.79%) *E. scrobiculatus* unigenes. They were annotated with the Pfam, SwissProt, KEGG, String, COG, KOG, NOG, and GO databases, respectively (Supplementary Figure S2). A total of 17,747 (82.58%) of the 21,491 unigenes were annotated based on the homology and matches to the aforementioned databases. In addition, 14,925 (69.45%) of the 21,491 unigenes were annotated against the NCBI NR protein database. Homology searches revealed that the *E. scrobiculatus* midgut transcriptomes had the highest similarity (54.41%) with the sequences from *D. ponderosae* followed by *Tribolium castaneum* (17.83%; Supplementary Figure S3).

**FIGURE 1:**
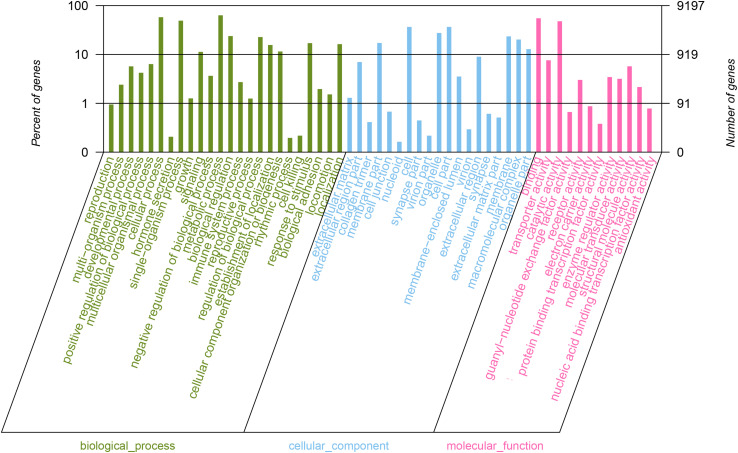
Functional annotation of all unigenes based on gene ontology (GO) categorization. GO analysis was performed at the level for three main categories (cellular component, molecular function, and biological process).

A Blast2GO search of the NR database assigned the GO terms ‘biological process’, ‘cellular component’ and ‘molecular function’ categories to 9,197 (15.61%) of the *E. scrobiculatus* unigenes. The cellular and metabolic process subcategories were the most highly represented within the biological process category. The cell and cell parts subcategories were highly abundant in the cellular component category. The most abundant unigenes in the molecular function category were associated with ‘binding’ and ‘catalytic activity’ (Figure 1).

### Identification of Putative *E. scrobiculatus* PCWDE Candidates

We identified 25 unigenes encoding putative *PCWDEs* in the *E. scrobiculatus* midgut transcriptomes and the previous transcriptome (GenBank accession number: SRX719565). *EscrGH45-1*,*EscrGH28-1*, *EscrGH28-2* and *EscrGH28-3*were identified in this previous transcriptome (SRX719565), and other genes were identified in the midgut transgene (SRP070604). These unigenes were verified by a BLASTx homology search. We selected the top matches for each unigene (Tables 1, 2 and Supplementary Table S3) and named them according to the Escr-enzyme-number format (named from 1–n, where n is the number of each enzyme family). Two genes (*EscrGH48-3* and *EscrCE8-3*) obtained complete open reading frames by 3’RACE and the 3’RACE primers are shown in Supplementary Table S4.

**TABLE 1:** Best BLASTX matches of *E. scrobiculatus* putative cellulases (GH48, GH45, and GH9).

Number	Unigene length (bp)	ORF length (aa)	Complete ORF	Signal peptide AA	FPKM	Best BLASTX match					
						Name	Acc. number	Species	Score	E-value	Identity (%)
EscrGH48-1	2033	637	YES	1–17	345.209	cellulose 1,4-beta-cellobiosidase	CAH25542.1	*Otiorhynchus sulcatus*	990	0.0	80
EscrGH48-2	3211	616	YES	1–19	70.988	glycoside hydrolase family protein 48	XP_019764094.1	*Gastrophysa viridula*	696	0.0	54
EscrGH48-3	2240	618	YES	1–20	94.72	glycoside hydrolase family protein 48	XP_019756174.1	*Dendroctonus ponderosae*	1030	0.0	79
EscrGH48-4	1864	607	YES	1–19	2.562	glycoside hydrolase family protein 48	XP_019757752.1	*Dendroctonus ponderosae*	1013	0.0	77
EscrGH48- 5	1997	637	YES	-	5676.389	glycoside hydrolase family protein 48	ADU33252.1	*Sitophilus oryzae*	1017	0.0	77
EscrGH45-1	1043	237	YES	1–18	93.059	endoglucanase-like	XP_019771465.1	*Dendroctonus ponderosae*	357	5e-121	76
EscrGH45-2	1027	247	YES	1–17	4276.963	endoglucanase-like	XP_019757792.1	*Dendroctonus ponderosae*	381	2e-130	80
EscrGH45-3	1026	236	YES	1–17	4.285	endoglucanase-like	XP_019772608.1	*Dendroctonus ponderosae*	388	2e-133	83
EscrGH45-4	876	240	YES	1–16	182.884	endoglucanase-like	XP_019766961.1	*Dendroctonus ponderosae*	385	5e-133	72
EscrGH45-5	792	230	YES	1–20	8098.053	endoglucanase-like	XP_019754618.1	*Dendroctonus ponderosae*	363	7e-125	75
EscrGH9-1	1639	479	YES	1–20	3.151	endoglucanase 13-like	XP_018323591.1	*Agrilus planipennis*	473	1e-159	50

**TABLE 2:** A comparison of the number of plant cell wall-degrading enzymes identified from *E. scrobiculatus* and other beetles.

		Cellulases			Pectinases		
		GH48	GH45	GH9	GH28	CE8	PL4
Tenebrionidae	*Tribolium castaneum*	–	–	1	–	–	
Curculionidae	*Dendroctonus ponderosae*	6	9	–	16	7	4
	*Sitophilus oryzae*	2	5	–	6	5	
	*Rhynchophorus ferrugineus*	2	1	2	3	3	
	*Eucryptorrhynchus scrobiculatus*	5	5	1	7	3	4
Chrysomelidae	*Diabrotica virgifera*	2	10	–	10	–	
	*Gastrophysa viridula*	3	1	–	7	–	
	*Chrysomela tremula*	2	2	–	9		
	*Phaedon cochleariae*	–	7	–	9	–	
	*Leptinotarsa decemlineata*	3	7	–	11	–	
	*Agrilus planipennis*		–	1	7		
Cerambycidae	*Apriona japonica*	–	5	–	2	–	
	*Acalolepta longipennis*	–	1	1	–	–	
	*Anoplophora glabripennis*	2	2	1	16		

**T castaneum*, *A. japonica*, *A. longipennis, and R. ferrugineus* (Antony et al., 2017); *D. ponderosae*, *T. castaneum*, *A. glabripennis*, and *A. planipennis* (McKenna et al., 2016); *D. virgifera* (Eyun et al., 2014); *G. viridula*, *C. tremula*, and *L decemlineata* (Pauchet et al., 2010); *P. cochleariae* (Roy et al., 2012); *S. oryzae* (Pauchet et al., 2010).*

Of the 25 PCWDEs, 11 were grouped into cellulolytic enzymes (GH9, GH45, and GH48) and 14 were annotated as pectolytic enzymes (GH28, PL4, and CE8) (Table 1 and Supplementary Table S4). Cellulose digestion involves several enzymes in the GH superfamily. Our analysis of the midgut transcriptomes of *E. scrobiculatus* disclosed five GH48 family genes. All but one unigene (*EscrGH48-4*) had complete ORFs. Four unigenes comprised predicted signal peptide (SP) sequences. EscrGH48-5 matched *Sitophilus oryzae* GH48, EscrGH48-1 matched *Otiorhynchus sulcatus* GH48 (CAH25542.1), *EscrGH48-2* and *EscrGH48-4* matched *Gastrophysa viridula* GH48, and EscrGH48-4 shared 50% similarity with *Leptinotarsa decemlineata* GH48. The FPKM values for *EscrGH48-5* and *EscrGH48-1* were 5,676.389 and 345.209, respectively. Thus, they were highly expressed genes (Table 1). Members of the GH45 class of CAZy enzymes have endoglucanase activity and various uncharacterized functions (Busch et al., 2019). Five candidates of this class have been identified (*EscrGH45-1*–*EscrGH45-5*). All EscrGH45 sequences matched the GH45 enzymes of *D. ponderosae* with >70% similarity (Table 1). GH9 class enzymes have endoglucanase activity. Here, we identified *EscrGH9-1* which shared 55% homology with *Anoplophora glabripennis* GH9 (XP_018568194.1 or AGLA010313; https://i5k.nal.usda.gov/).

Seven GH28 unigenes (*EscrGH28-1*–*EscrGH28-7*), encoding putative pectin-degrading polygalacturonases were identified in the InterPro program (Supplementary Table S4). Four GH28 unigenes were highly expressed in midgut transcription (FPKM > 10). In other insects, their homologs function as polygalacturonases. *EscrGH28-6* resembled the GH28 reported for *S. oryzae*. The others were similar to GH28 from *D. ponderosae* (Supplementary Table S4). Enzymes of the CE8 class function as pectin methylesterases (PMEs) and degrade the pectin network. We putatively identified three CE8 unigenes. *EscrCE8-1* and *EscrCE8-2* shared high sequence similarity with *S. oryzae* PME. The *EscrCE8-3* sequence most closely resembled the CE8 from *S. oryzae* (Supplementary Table S4). *EscrCE8-2* shared 59% similarity with *S. oryzae* pectin methylesterase. The latter is the only beetle pectinase whose structure has been elucidated and reported (Teller et al., 2014). Four unigenes were assigned to PL4. *EscrPL4-1* shares 68% similarity with *D. ponderosae* PL4. *EscrPL4-2* sequences matched the PL4 enzymes in *D. ponderosae* with >72% homology. *EscrPL4-3* and *EscrPL4-4* shared high sequence similarity with *D. ponderosae* PL4 (Supplementary Table S4).

### Phylogenetic Analysis of *E. scrobiculatus* PCWDEs

#### Candidate GH48 Genes

**FIGURE 2:**
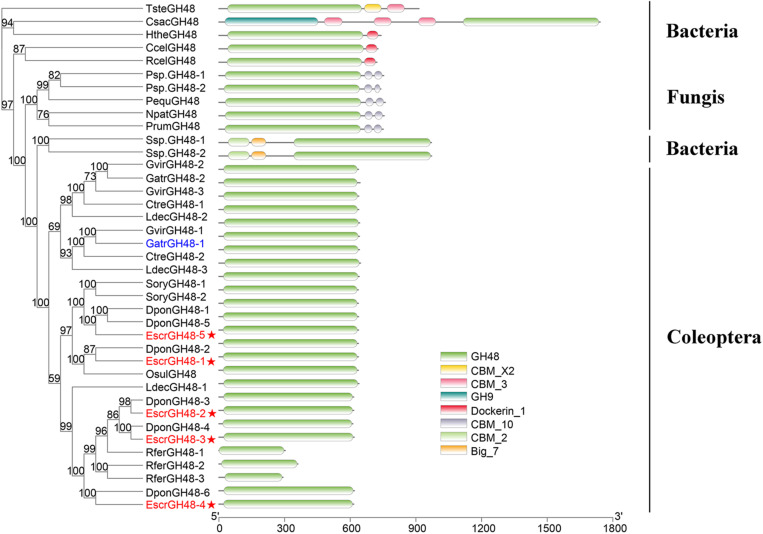
Comparison of amino acid sequences and domain structures in *E. scrobiculatus* and glycosyl hydrolase family 48 (Family GH48) members. The scale indicates distance (number of amino acid substitutions per site). The *E. scrobiculatus* genes are shown in red and marked with an asterisk. Members of fungi, bacteria, and insects were temporarily grouped. GH48, catalytic domain of glycosyl hydrolase family 48; GH9, catalytic domain of glycosyl hydrolase family 9; CBM 3, 10, cellulose binding domain; Big7, Bacterial Ig domain: CBM X2, Carbohydrate binding domain X2. Amino acid sequences used for the tree are listed in Supplementary Table S5.

A phylogenetic tree was constructed from the catalytic domain sequences of five of the GH48 family genes and those of fungi, bacteria, and Coleoptera. The *E. scrobiculatus* GH48 sequences had high homology to the endoglucanases from *D. ponderosae*. The GH48 genes from the Coleoptera had only one GH48 domain. However, the GH48 genes from bacteria and fungi had other functional domains in addition to GH48 (Figure 2).

#### Candidate GH45 Genes

**FIGURE 3:**
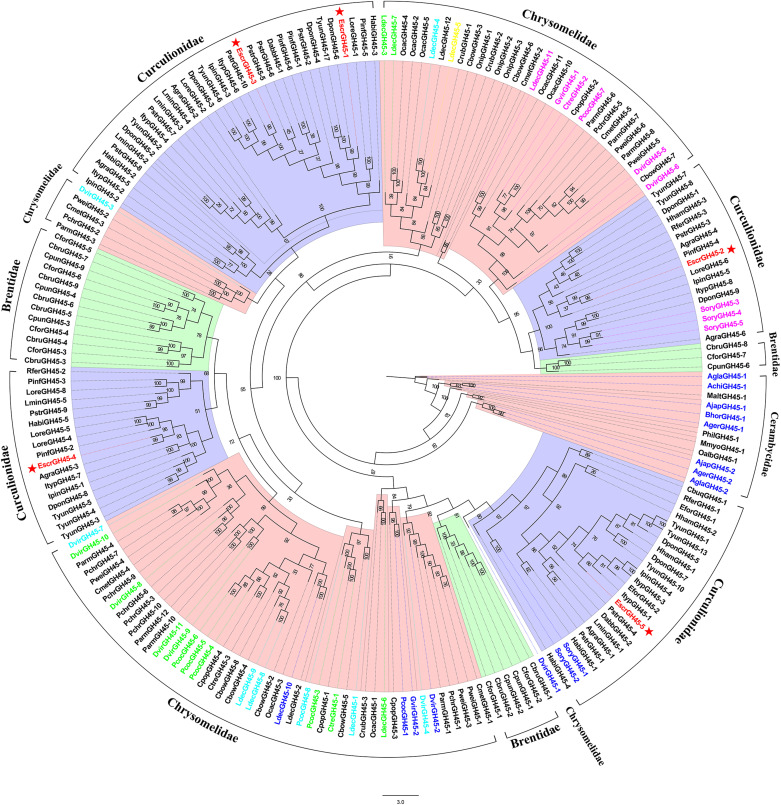
Comparison of amino acid sequences in *E. scrobiculatus* and glycosyl hydrolase family 45 (Family GH45) members. The *E. scrobiculatus* genes are shown in red and marked with an asterisk. Amino acid sequences used for the tree are listed in Supplementary Table S5. GH45s characterized to date are color-coded based on their activity; blue = endo-β-1,4-glucanases; green = endo-β-1,4-glucanases and (gluco)mannanases; yellow = endo-β-1,4-glucanases and endo-β-1,4-xyloglucanases; magenta = endo-β-1,4-xyloglucanases; cyan = no activity detected.

A phylogenetic tree was built using all GH45 families and those of the Coleoptera. The EscrGH45 family genes clustered with those of the Curculionidae. The GH45 family genes from Curculionidae were grouped into four clusters. The EscrGH45 family genes were distributed into four clusters (Figure 3). *EscrGH45-2* clustered with the GH45 gene of *S. oryzae*, which has been verified to have endo-β-1,4-xyloglucanases activity. At the same time, *EscrGH45-5* clustered with the GH45 gene of *S. oryzae*, which has been verified to have endo-β-1,4-glucanases activity.

#### Candidate GH9 Genes

**FIGURE 4:**
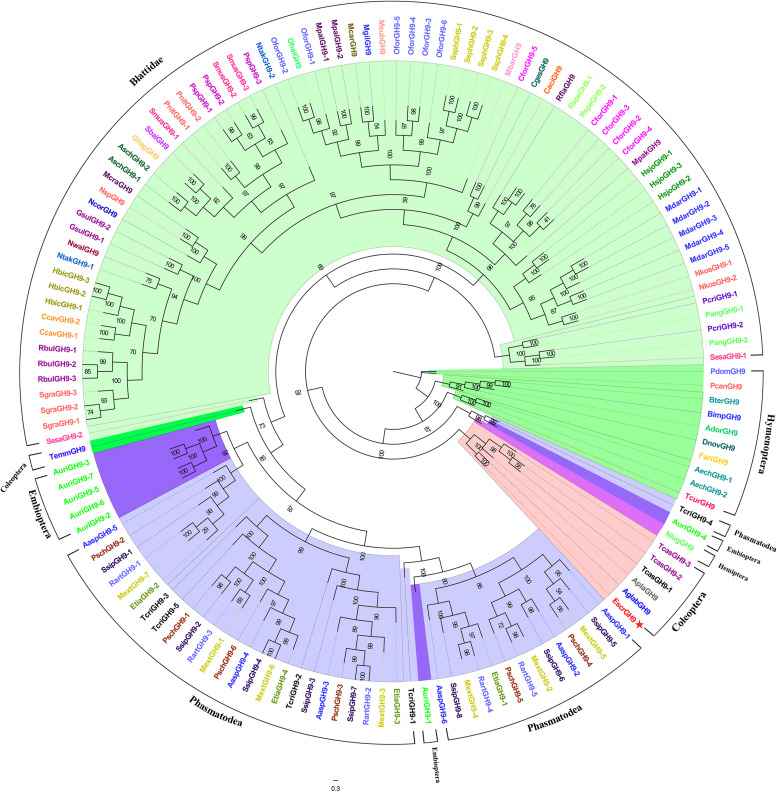
Comparison of amino acid sequences in *E. scrobiculatus* and glycosyl hydrolase family 9 (Family GH9) members. The scale indicates distance (number of amino acid substitutions per site). The *E. scrobiculatus* genes are shown in red and marked with an asterisk. Amino acid sequences used for the tree are listed in Supplementary Table S5.

Comparatively few GH9 family genes were found in beetles. We used GH9 sequences from *E. scrobiculatus* and other insects (Coleoptera, Hymenoptera, Hemiptera, Phasmatodea, Blattodea, Embioptera, and Orthoptera) to build a phylogenetic tree. *EscrGH9-1* had the highest homology with the *A. glabripennis* protein. The Coleoptera sequences were clustered into one group (Figure 4).

#### Candidate GH28 Genes

**FIGURE 5:**
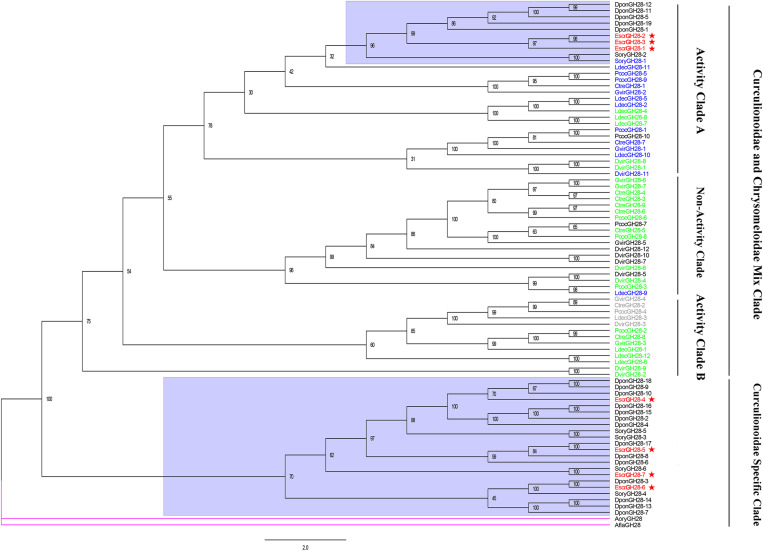
Comparison of amino acid sequences in *E. scrobiculatus* and glycosyl hydrolase family 28 (Family GH28) members. The *E. scrobiculatus* genes are shown in red and marked with an asterisk. Amino acid sequences used for the tree are listed in Supplementary Table S5. GH28 genes characterized to date are color-coded based on their activity; blue = activity on pectin; green = no activity detected; gray = activity toward tri-galacturonic acid.

We performed a phylogenetic analysis on our candidate GH28 family genes and those from the phytophagous beetles (Curculionidae and Chrysomelidae) by a Maximum Likelihood analysis where two fungal GH28 genes from Aspergillaceae were taken as an outgroup. All GH28 genes of the Curculionidae specific clade were not characterized, and *EcrGH28-4*, *EcrGH28-5*, *EcrGH28-6*, and *EcrGH28-7* belong to this clade. Remarkably, the Curculionidae and Chrysomelidae clade contained three distinct subclades: one with almost all Chrysomelidae GH28 genes and *SoryGH28-1* active on pectin (active clade A), one with five genes active only on pectic trimers (active clade B), and the third including all Chrysomelidae GH28 genes (except *LdecGH8-9*) with non-active on pectin or pectic trimers (non-active clade). *EcrGH28-1*, *EcrGH28-2*, and *EcrGH28-3* belong to the active clade A (Figure 5).

#### Candidate CE8 Genes

**FIGURE 6:**
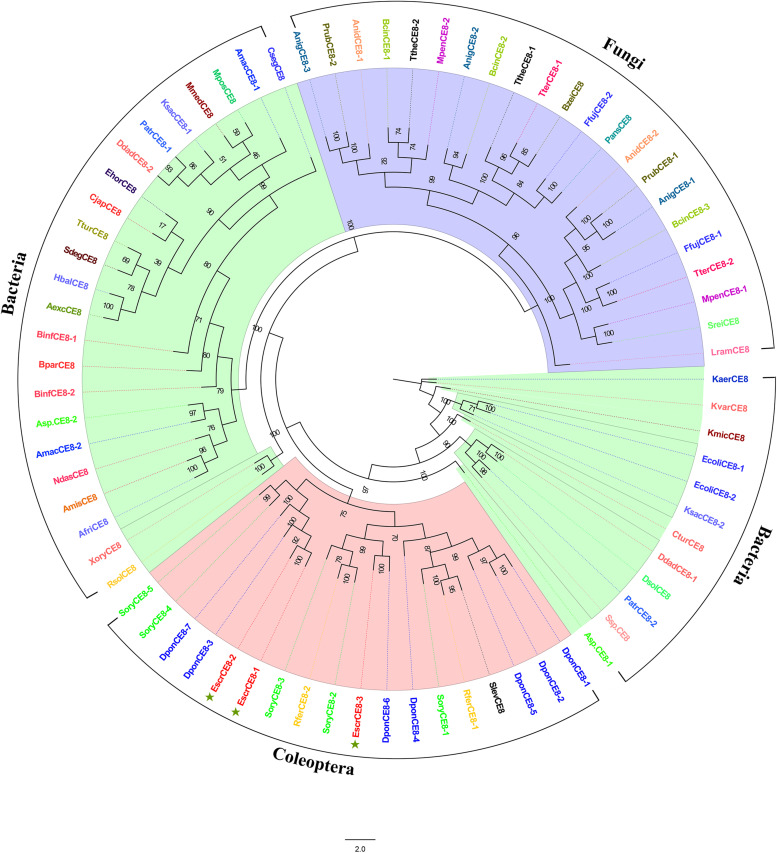
Comparison of amino acid sequences in *E. scrobiculatus* and glycosyl hydrolase family CE8 (Family GE8) members. The scale indicates distance (number of amino acid substitutions per site). The *E. scrobiculatus* genes are shown in red and marked with an asterisk. Amino acid sequences used for the tree are listed in Supplementary Table S5.

We identified three putative CE8 unigenes. *EscrCE8-1* and *EscrCE8-2* shared high sequence similarity with *S. oryzae* PME (ADU33262.1). EscrCE8-3 was most similar to CE8 from *S. oryzae* (ADU33260.1; Table 3). *EscrCE8-2* shared 59% similarity with *S. oryzae* pectin methylesterase. It is the only beetle pectinase whose structure has been elucidated and reported (Teller et al., 2014). The CE8 phylogenetic tree revealed that the CE8 genes of the Curculionidae shared similarity with those of bacteria (Figure 6).

**TABLE 3:** The function of plant cell wall-degrading enzymes has been verified in beetles.

Gene	Species	NCBI number	NAL number
GH48	*Gastrophysa atrocyanea*	AB241611	
GH45	*Apriona germari*	AY451326	
GH45	*Batocera horsfieldi*	KP325107	
GH45	*Apriona germari*	AY162317	
GH45	*Anoplophora malasiaca*	JN581572	
GH45	*Anoplophora glabripennis*		AGLA005419
GH45	*Anoplophora glabripennis*		AGLA005420
GH45	*Diabrotica virgifera virgifera*	JQ755253	
GH9	*Anoplophora malasiaca*	AGLA010313	
GH28	*Anoplophora glabripennis*		AGLA010095
GH28	*Anoplophora glabripennis*		AGLA010096
GH28	*Anoplophora glabripennis*		AGLA010097
GH28	*Anoplophora glabripennis*		AGLA010099
GH28	*Anoplophora glabripennis*		AGLA002350
GH28	*Anoplophora glabripennis*		AGLA025090
GH28	*Leptinotarsa decemlineata*	ADU33345.1-ADU33351.1	
GH28	*Leptinotarsa decemlineata*	MH892458- MH892461	
GH28	*Chrysomela tremula*	ADU33285.1-ADU33286.1	
GH28	*Chrysomela tremula*	MH892457	
GH28	*Phaedon cochleariae*	HE962202.1-HE962208.1	
GH28	*Diabrotica vir. virgifera*	AFI56547.1	
GH28	*Diabrotica vir. virgifera*	MH892463-MH892472	
GH28	*Sitophilus oryzae*	ADU33246.1-ADU33250.1	
CE8	*Sitophilus oryzae*	HM175754.1- HM175755.1	

*National Agricultural Library (NAL) (https://i5k.nal.usda.gov/).*

### Tissue Expression Analysis of *E. scrobiculatus* PCWDEs

**FIGURE 7:**
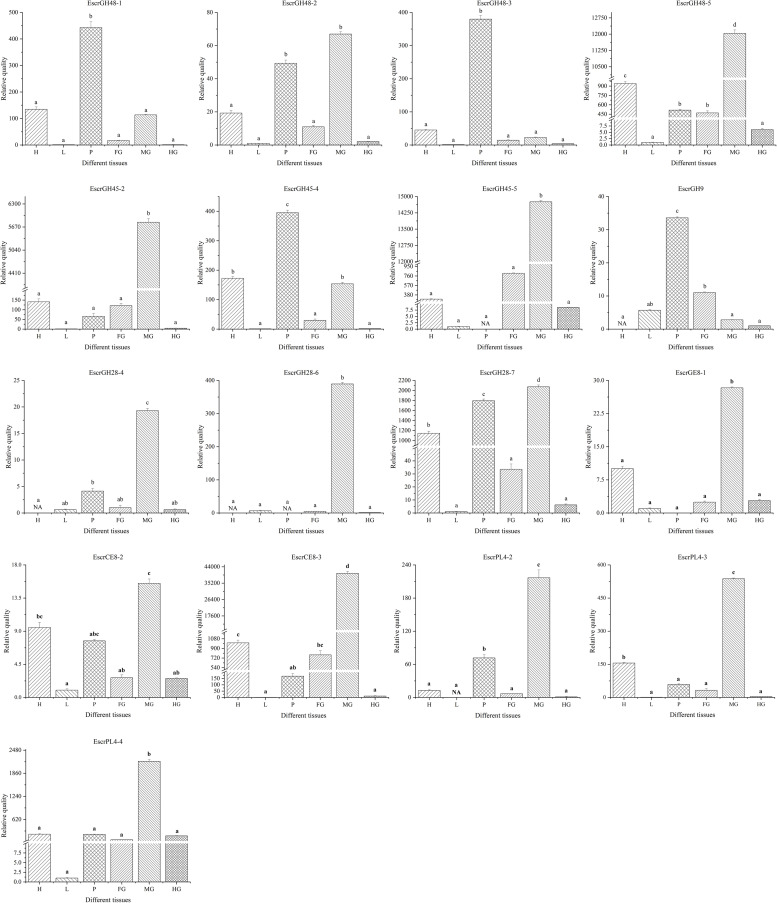
Plant cell wall-degrading enzymes (PCWDEs) transcript levels in different tissues of *E. scrobiculatus*. NA means the transcript level too low to measure.

The specificity of the PCWDE primer was determined by RT-PCR. Fluorescence RT-qPCR was conducted to determine the expression patterns of the PCWDEs in various tissues. The expression levels of 13 genes were significantly higher in the midgut than the other tissues. These genes included three CHs (*EscrGH45-2*, *EscrGH45-2*, and *EscrGH45-5*) belonging to the cellulases, and eight genes (*EscrCE8-1*, *EscrCE8-3*, *EscrGH28-4*, *EscrGH28-6*, *EscrGH28-7*, *EscrPL4-2*, *EscrPL4-3*, and *EscrPL4-4*) belonging to the pectinases. *EscrGH9-1*, *EscrGH45-4*, *EscrGH48-1*, and *EscrGH48-5* were highly expressed in the proboscis than the other tissues. *EscrCE8-2* and *EscrGH48-2* were upregulated in the midgut but their expression levels there did not significantly differ from those in the other tissues (Figure 7).

## Discussion

Twenty-five genes encoding PCWDEs and belonging to the glycoside hydrolases (GHs), polysaccharide lyases (PLs), and carbohydrate esterases (CEs) of CAZy enzymes were identified in the *E. scrobiculatus* midgut transcriptome (Table 2). A BLAST search in the NR and Swiss-Prot uniprot databases revealed that these genes were annotated as GH48, GH45, GH28, GH9, PL4, and CE8. Most of the candidate genes shared a high homology with those of *D. ponderosae* and *S. oryzae*. We performed statistics on the relevant PCWDE genes that had been functionally verified (Table 3). In order to predict the evolutionary relationship between these genes of *E. scrobiculatus* and related genes of other species, we conducted phylogenetic and domain analysis. A tissue-specific expression analysis of the gut, the main digestive organ in insects, disclosed relatively higher expression levels of 17 of the 25 identified PCWDEs. Our study determined the types and relative expression levels PCWDEs of *E. scrobiculatus*. These PCWDEs may be used for pest control of E. scrobiculatus and biofuels.

Previous studies have shown that endogenous cellulase of insects includes GH48, GH45, GH9, and GH5 (Pauchet et al., 2010; Calderón-Cortés et al., 2012; Fischer et al., 2013). Here, 11 genes belonging to GH48, GH45, and GH9 families were identified. However, no representatives of the GH5 family gene were detected (Table 1).

GH48 commonly occurs in cellulolytic bacteria and GH48 genes have cellulase activity (Berger et al., 2007). GH48 genes were identified in two fungal species (*Piromyces equi* and *Piromyces sp.*) (Steenbakkers et al., 2002). The GH48 family genes in *Orpinomyces sp.* and *Neocallimastix patriciarum* were also confirmed to have cellulase activity (Tzi-Yuan et al., 2011; Morrison et al., 2016). GH48 family genes have been identified in the superfamilies Curculionoidea and Chrysomeloidea of the Coleoptera (Fujita et al., 2006; Pauchet et al., 2010, 2014; Keeling et al., 2012; Eyun et al., 2014; McKenna et al., 2016; Antony et al., 2017). GH48 family genes have cellulase activity in bacteria and fungi. However, there is no evidence to indicate that insect GH48 family genes can break down cellulosic polysaccharides. GH48 family genes *GatrGH48-1* and *GatrGH48-2* (Figure 3) were isolated from *Gastrophysa atrocyanea*. Chitinase activity was associated with *GatrGH48-1* (active phase-associated proteins (APAP) I). However, it has neither glucanase nor cellobiohydrolase activity (Fujita et al., 2006). No cellulase activity was found for the GH48 gene of Otiorhynchus *sulcatus* (Edwards et al., 2002). A domain structure analysis of the GH48 family genes by Pfam^5^ demonstrated that insect GH48 family genes have only one GH48 domain. The GH48 family genes of the Coleoptera share considerable amino acid sequence homology with those of bacteria and fungi (Figure 2). Nevertheless, the GH48 family genes in bacteria and fungi are constituents of complex proteins with multiple functional domains. The lack of other domains in *GatrGH48-1* may account for the fact that it has no cellulase activity. EscrGH48 has the same domain structure as *GatrGH48-1*. Thus, we speculate that the *EscrGH48* gene has chitinase activity and that the GH48 family of *E. scrobiculatus* does not have cellulase activity and does not degrade plant cells. The four genes of the GH48 family we quantitatively tested were highly expressed in the proboscis and midgut. The relative expression levels of *EscrGH48-1* and *EscrGH48-3* in the proboscis were significantly different from those in the other tissues. At the same time, the relative expression level of *EscrGH48-5* in the midgut was significantly different from that in other tissues. We speculate that the proboscis and midgut are both organs that digest chitin.

Previous research showed that phytophagous beetles presumably obtained GH45 genes from fungi through an HGT event (Busch et al., 2019). The GH45 family genes were described for the Coleopteran superfamilies Chrysomeloidea and Curculionoidea. The GH45 family of Chrysomelidae gene function has been differentiated and now has cellulase, xyloglucanase, and glucomannanase activity (Busch et al., 2019). To study the evolutionary relationship of the beetle’s GH45 genes, we constructed a maximum-likelihood phylogenetic tree using the GH45 family genes of Coleoptera (Pauchet et al., 2010; Eyun et al., 2014; Antony et al., 2017; Busch et al., 2019). This phylogenetic tree showed that the EscrGH45 gene was localized to four distinct groups (Figure 3). In the cluster of GH45 genes including EscrGH45-5, SoryGH45-1, and SoryGH45-2 of *S. oryzae* of Curculionidae have endo-β-1,4-glucanase and endo-β-1,4-xyloglucanase activity (Busch et al., 2019). The FPKM value of GH45-5 in the midgut transcriptome was ≤ 8,000. We speculate that EscrGH45-5 has cellulase activity. SoryGH45-3, SoryGH45-4, and SoryGH45-5 can only break down xyloglucan rather than cellulose. EscrGH45-2 is highly homologous with these genes. The FPKM of *EscrGH45-2* in the midgut transcriptome was 4,000. Thus, *EscrGH45-2* may have xyloglucanase activity. As more GH45 genes in weevil are functionally validated, these GH45 genes may have three enzymatic activities (endo-β-1,4-glucanase, endo-β-1,4-xyloglucanase and mannanase). The RT-qPCR analysis revealed that the relative expression levels of *EscrGH45-2* and *EscrGH45-5* in the midgut were significantly higher than other tissues. The proboscis presented with the highest expression levels of *EscrGH45-4*. We speculate that cellulose is digested in the proboscis and midgut in *E. scrobiculatus*. Initially, the GH9 gene was thought to be absent in the chrysomelid and curculionid superfamilies (Watanabe and Tokuda, 2010; Calderón-Cortés et al., 2012; Fischer et al., 2013). The GH9 family genes were considered lost in the ancestors common to Chrysomelidae and Curculionidae (Eyun et al., 2014). Recently, however, the GH9 gene was discovered in the two aforementioned superfamilies (Antony et al., 2017; Busch et al., 2018). The GH9 genes were distributed in all insect orders examined thus far. A common ancestor of the insects may have borne GH9 cellulase genes that were vertically transferred to extant (descendant) insects (Fischer et al., 2013). In the phylogenetic tree, the GH9 genes of the Coleoptera were grouped into one cluster and *EscrGH9-1* was highly homologous with *AglaGH9*. The latter harbored no endo-acting enzymatic activity for carboxymethylcellulose (McKenna et al., 2016). The FPKM for EscrGH9 in the midgut transcriptome was 3.151. The relative expression levels of *EscrGH9-1* were low in all six insect tissues. Of the Curculionidae that have been studied, none presented with the GH9 gene. Therefore, the GH9 gene function has been lost or replaced in these insects. Thus, *EscrGH9-1* may lack cellulase activity and may not be a plant cell-degrading enzyme.

Insect pectinases comprise rhamnogalacturonan lyases, pectin methylesterases, and polygalacturonases which belong to the multi-gene carbohydrate polysaccharide lyase family (PL4), esterase family 8 (CE8), and glycosyl hydrolase family 28 (GH28), respectively (Pauchet et al., 2010; Calderón-Cortés et al., 2012; Kirsch et al., 2016). Advances in sequencing technology and bioinformatic analysis have disclosed that endogenous GH28 is ubiquitous among insect taxa (Calderón-Cortés et al., 2012). Kirsch et al. (2014) indicated that three independent HGT events of GH28 genes occurred during the evolution of phytophagous beetles, one of which was retained by most descendent Curculionoidea (Kirsch et al., 2014). Phytophagous beetles acquired genes encoding active GH28 genes via HGTs, and these HGTs promoted their capacity to utilize plant material as a primary source of food (Kirsch et al., 2014). Most of the GH28 family genes identified in *A. glabripennis* of the Cerambycidae were active against at least one homogalacturonan polymer. As these gene functions are highly complementary, *A. glabripennis* efficiently decomposes pectineus homogalacturonan polymers. However, no other pectinase was identified in *A. glabripennis* (McKenna et al., 2016). Up to 19 GH28 family genes were identified in *D. ponderosae* (Eyun et al., 2014). At the same time, PL4 and CE8 were also identified in *D. ponderosae* (Pauchet et al., 2010). In addition to pectinase activity and tri galacturonase activity, the high number of catalytically inactive phytophagous GH28 proteins may act as “decoy” targets for GH28 inhibitory proteins, thus protecting the active GH28 proteins from inhibition (Kirsch et al., 2014). Here, seven GH28 genes were identified in *E. scrobiculatus* and four of them were localized to the midgut transcriptome. The FPKM for *EscrGH28-4* and *EscGH28-7* in the midgut transcriptome were 179.462 and 638.290, respectively. Phylogenetic analysis revealed that EscrGH28, DponGH28, and SoryGH28 genes were grouped into two distinct subclades based on the beetle’s GH28 protein (Figure 5). We speculate that the function of EscrGH28 genes may have differentiated. Kirsch et al. (2016) study indicated that the digestion of complex pectin polysaccharides in *S. oryzae* required CE8 family genes in addition to GH28 family genes (Kirsch et al., 2016). The function of these CE8 family genes has been verified, two of which have pectinase activity (Shen et al., 1999; Kirsch et al., 2016). Three CE8 genes were also identified *E. scrobiculatus* and the FPKM for *EscCE8-1* was 13,453.841. To date, CE8 genes have only been found in weevils and in no other animals. The origin of weevil CE8 genes can be best explained by a HGT event from bacteria to weevils (Evangelista et al., 2015; Kirsch et al., 2016). In addition to the families of GH28 and CE8 genes, five PL4 family genes were described for *D. ponderosae.* They were putative rhamnogalacturonan lyases which constitute another class of pectolytic enzymes (Antony et al., 2017). Four PL4 family genes were identified in *E. scrobiculatus* (Supplementary Table S4). Except for EscPL4-3, the FPKMs of the PL4 family genes were ≥ 250. To date, the PL4 family of genes have only been identified in the Curculionidae. The RT-qPCR analysis was performed on the nine putative pectinase genes with comparatively high FPKMs (*EscrPL4-2*, *EscrPL4-3*, *EscrPL4-4*, *EscrCE8-1*, *EscrCE8-2*, *EscrCE8-3*, *EscrGH28-4*, *EscrGH28-6*, and *EscrGH28-7*). The relative expression level of those genes except for *EscrGE8-2* in the midgut was significantly higher than that in other tissues. The midgut is the main digestive organ of insects, and high expression of these genes also indirectly indicates that these genes are related to digestion. In summary, we speculate that the GH28, CE8, and PL4 genes in *E. scrobiculatus* synergistically break down the complex polysaccharide of pectin. The functions of the PCWDEs examined in the present study were neither determined nor validated. In future research, we will verify the functionality of the PCWDEs. The potential of cellulase application in RNAi-medicated pest control strategies has been demonstrated by silencing a termite endogenous cellulase (Zhou et al., 2008). Plant cellulose can be digested by cellulase to produce the bioenergy source cellulosic ethanol (Willis et al., 2017). With the development of genetic engineering of bioenergy, elucidation of the functions of the major PCWDEs will be highly valuable.

Here, we identified 25 unigenes encoding the GH family enzymes GH48, GH45, GH38, GH28, and GH16), four unigenes encoding PL4 family enzymes, and three unigenes encoding CE family enzymes in the *E. scrobiculatus* transcriptome. Quantitative expression, phylogenetic analyses, and FPKMs identified the cellulases (*EscrGH45-2* and *EscrGH45-5*) and pectinases (*EscrGH28-7*, *EscrCE8-3*, and *EscrPL4-4*) that were the most highly expressed in *E. scrobiculatus*. Thence, the enzymatic activities of the PCWDEs were predicted.

As *E. scrobiculatus* is a major pest of *A. altissima*, identification of the PCWDEs has practical applications in *E. scrobiculatus* pest management. We identified the PCWDEs, performed a phylogenetic analysis on them, determined their relative expression patterns and levels in various insect tissues. The present work has reference significance for the study of other plant cell wall degrading enzymes in weevils.

## Data Availability Statement

The datasets transcriptome for this study can be found in in the NCBI SRA database under GenBank accession number SRP070604.

## Author Contributions

PG and JW conceived and designed the experiments. PG, ZL, and JW participated in regular discussions regarding the design of the laboratory experiments and analysis of the sequencing data. PG performed the experiments and analyzed the data. JW obtained funding. PG contributed to the writing of this article. All authors read and approved the final manuscript.

## Conflict of Interest

The authors declare that the research was conducted in the absence of any commercial or financial relationships that could be construed as a potential conflict of interest.

## Footnotes

1 https://github.com/jstjohn/SeqPrep

2 https://github.com/najoshi/sickle

3 http://trinityrnaseq.sourceforge.net/

4 http://www.ncbi.nlm.nih.gov/gorf/gorf.html

5 http://pfam.xfam.org/
