# Supplementary material for: Expression Profiling of Plant Cell Wall-Degrading Enzyme Genes in Eucryptorrhynchus scrobiculatus Midgut
Source: Front Physiol. 2020 Sep 4;11:1111. doi: 10.3389/fphys.2020.01111 (PMC7500146; doi:10.3389/fphys.2020.01111)
Supplement: Supplementary file 1 [file Table_1.DOCX]

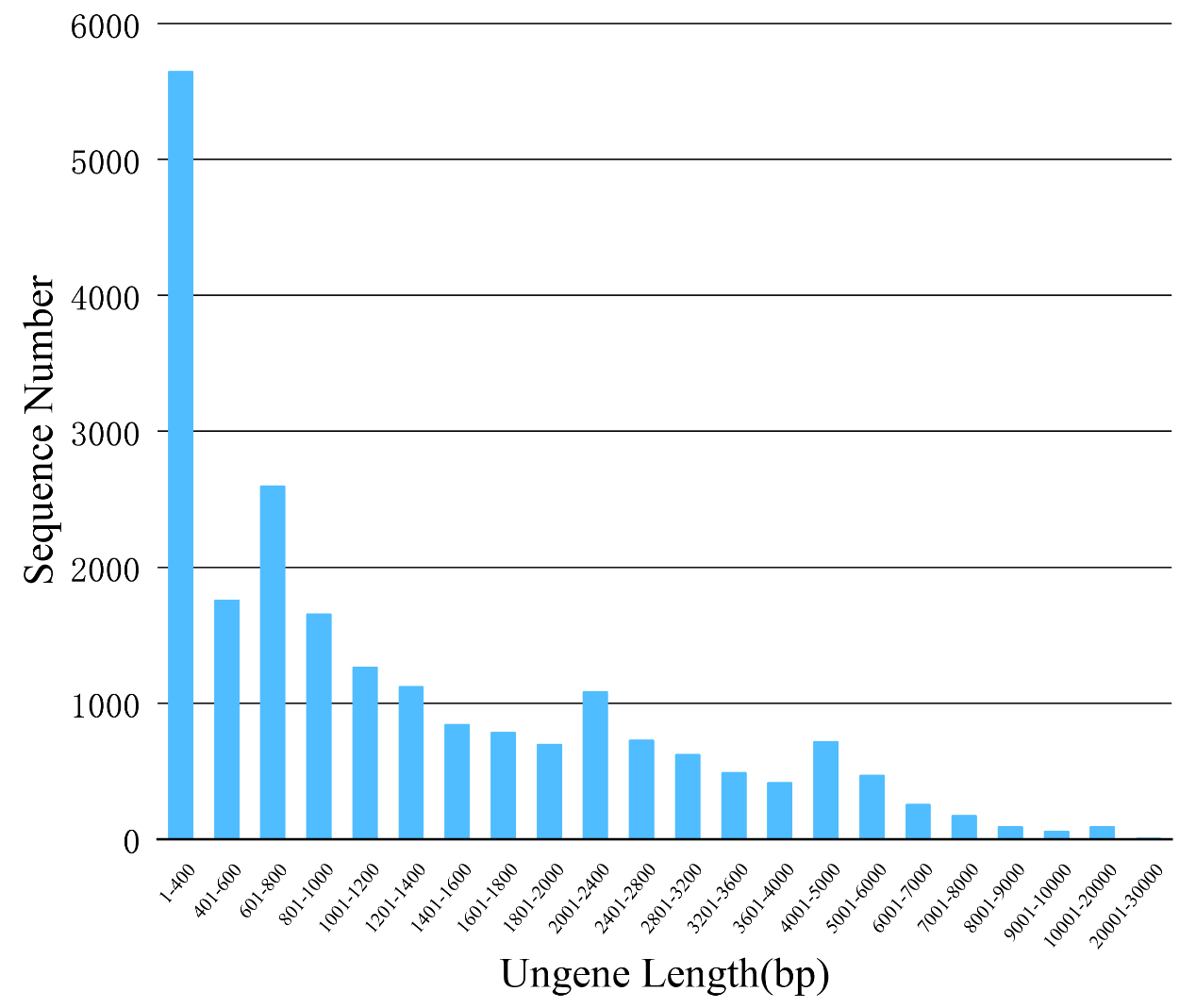
 **Supplementary figure S1. The size distribution of the unigenes from *E. scrobiculatus* assembled transcriptome**


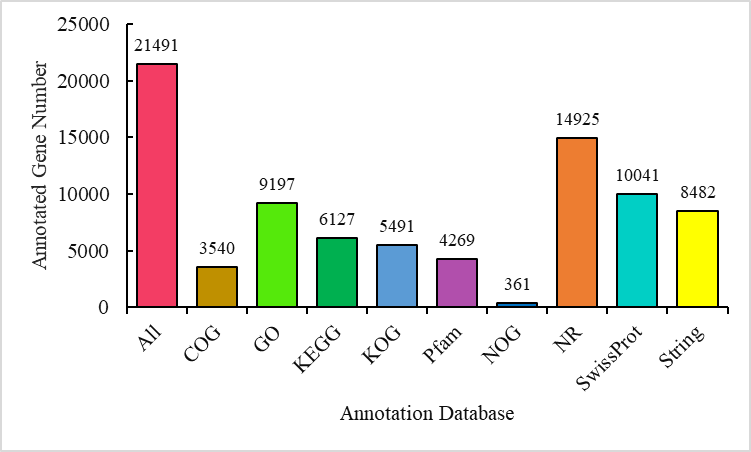


**Supplementary figure S2. Number of annotated genes of *E. scrobiculatu*s midgut transcriptome in various databases.**


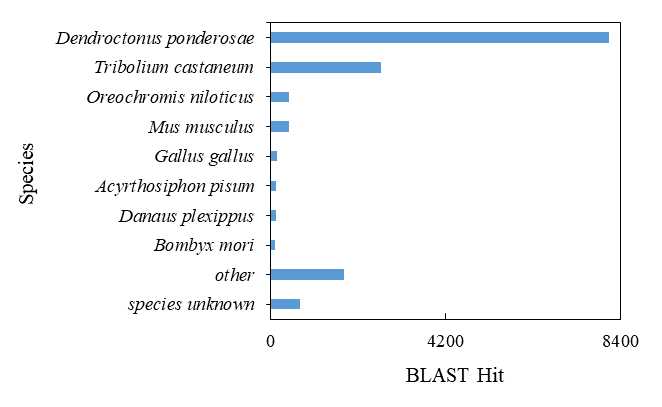


**Supplementary figure S3. *E. Scrobiculatus* midgut transcriptome ungenes aligned species and number.**

**Supplementary table S1.** An overview of the sequencing and assembly process.

| Item | **ESM1**  **ESM2**  **ESM3** | | **ESM1** | **ESM1**  **M** |
| --- | --- | --- | --- | --- |
| Total Raw Reads | 56,124,108  62528960  51476096 | | 62,528,960 | 51,476,096 |
| Total Base | 8,418,616,200 | | 9,379,344,000 | 7,721,414,400 |
| GC Content (%) | 41.29 | | 42.24 | 41.7 |
| Raw Reads Q30 Q30 Q30 (%) | 91.81 | | 92.17 | 91.95 |
| Raw Reads Q20 (%) | 96.57 | | 96.76 | 96.64 |
| Combined Trinity assembly of the male and female antennaltranscriptomestranscriptomestranscriptomestranscriptomestranscriptomestranscriptomestranscriptomestranscriptomestranscriptomestranscriptomes transcriptomestranscriptomestranscriptomes | | | | |
| Total Number | | **21,491** | | |
| Total Length | | 32,684,901 | | |
| Unigene N50 | | 2,736 | | |
| Mean Length | | 1,520.86 | | |

**Supplementary table S2.** Primers for fluorescence quantitative real-time PCR.

| **Name** | **Forward primer** | **Reverse primer** |
| --- | --- | --- |
| RPL13 | ACAGAAGGAAGCGTGTTGTGGTT | TTCCAGCCAGCCTCGTGTGA |
| EscrGH48-1 | GCATTGGCTGTTGGATGTTGACAA | CATCGCAAGTTGGCTGAGGAATTG |
| EscrGH48-2 | TGGACTGGAACCTACCCGAATGG | CGAGTGCCACATCAGCCTGTTC |
| EscrGH48-3 | AGGAACTCGTTATCCAGACAGGTAGA | GTCCACGCTCCAAGTCCACATT |
| EscrGH48-5 | TCTGGATCAGCTTCTGGACCTTCC | TCAGCATCAGGAGCAGCAGTGTA |
| EscrGH45-2 | CGCCGCAGCCGCTTTCATTA | ATCCAACTCCACCTCCTGGCATAG |
| EscrGH45-4 | AAGCCGTTACAACTTGCGACCTC | TGACTCACATCTGCTCCACCTGT |
| EscrGH45-5 | GAACGTGAACACTGGCGGAGATT | TGGAAGTTCGTTACACTCGGCATC |
| EscrGH9-1 | CAACGCCACCGACCACTTCATAG | TGTCGCAGGCAACGGTTCCA |
| EscrGH28-4 | AAGGGCGGCGAGAACGGAAT | CGAAGGCTGAGACGAATTGGATGG |
| EscrGH28-6 | CAAGACGATTGTGTTGCGGTGAA | CATGGATTGCGTTATTGGAGTGTTCT |
| EscrGH28-7 | GGACCTACAAGATGGAGCCACTCT | TCCTCTGCCGTCCCAGTACAATT |
| EscrPL4-2 | GCCAACTACTTCGTCAGCAGTCA | CTGAACCTCTGATTGATCCTCGTGAA |
| EscrPL4-3 | TGAGTGGTGCGGTAAGTGGACAA | GCCAGCCAGTGGATAAGCAACTATT |
| EscrPL4-4 | ATGGCAACACATCTACGGTCCTT | GTCCACTTACAATTCCGCTTACAGAG |
| EscrCE8-1 | CCCGCATGGGATCTCTACAAACAA | GCTTCGGTTCTGACTGCCAAGG |
| EscrCE8-2 | TGCTCCCGATACCGCTCCAAAT | ACACGCCAGGCACGTACAGAT |
| EscrCE8-3 | CAGACAGACGCAATACCGCTCTT | TGACCGTTGGCATTGGGATTCG |

**Supplementary table S3.** Primers for 3' RACE PCR.

| **Name** | **3'outer primer** | **3' inner primer** |
| --- | --- | --- |
| EscrGH48-4 | TACGGACAAAGCATTGGAAGTGC | CATCGGTTTTGGGAGCAGGC |
| EscrCE8-3 | GTTTTTGCTCCAAATACCGACAAC | ACTAACACCAACCGAAACCTGG |

**Supplementary table S4.** Best BLASTX matches of *E. scrobiculatus* putative pectinase genes (CH28, CE8 and PL4).

| **Number** | **Unigene length**  **(bp)** | **ORF length**  **(aa)** | **Complete**  **ORF** | **Signal**  **peptide**  **AA** | **FPKM** | **Best BLASTX match** | | | | | |
| --- | --- | --- | --- | --- | --- | --- | --- | --- | --- | --- | --- |
|  |  |  |  |  |  | **Name** | **Acc. number** | **Species** | **Score** | **E-value** | **Identity**  **(%)** |
| EscrCH28-1 | 1164 | 364 | YES | 1-17 | 360.962 | endopolygalacturonase | AEE62815.1 | *Dendroctonus ponderosae* | 542 | 0.0 | 72 |
| EscrCH28-2 | 1172 | 364 | YES | 1-17 | 144.260 | endopolygalacturonase | AEE62815.1 | *Dendroctonus ponderosae* | 538 | 0.0 | 71 |
| EscrCH28-3 | 1163 | 363 | YES | 1-17 | 15.480 | polygalacturonase-like | XP_019764759.1 | *Dendroctonus ponderosae* | 556 | 0.0 | 74 |
| EscrCH28-4 | 1301 | 371 | YES | 1-20 | 179.462 | polygalacturonase-like | XP_019756276.1 | *Dendroctonus ponderosae* | 461 | 4e-159 | 61 |
| EscrCH28-5 | 1155 | 367 | YES | 1-19 | 2.730 | endopolygalacturonase A-like | XP_019762278.1 | *Dendroctonus ponderosae* | 497 | 1e-173 | 64 |
| EscrCH28-6 | 1378 | 358 | YES | 1-20 | 60.179 | endopolygalacturonase | AEE63283.1 | *Dendroctonus ponderosae* | 537 | 0.0 | 73 |
| EscrCH28-7 | 1339 | 377 | YES | 1-17 | 638.290 | endopolygalacturonase I-like | XP_019761280.1 | *Dendroctonus ponderosae* | 493 | 1e-171 | 62 |
| EscrCE8-1 | 4587 | 383 | YES | 1-18 | 34.466 | pectin methylesterase | AEE62813.1 | *Dendroctonus ponderosae* | 491 | 2e-170 | 61 |
| EscrCE8-2 | 3827 | 385 | YES | 1-19 | 34.466 | pectin methylesterase | AEE62813.1 | *Dendroctonus ponderosae* | 552 | 0.0 | 68 |
| EscrCE8-3 | 1408 | 374 | YES | 1-21 | 13453.841 | pectin methylesterase | AEE62090.1 | *Dendroctonus ponderosae* | 520 | 0.0 | 72 |
| EscrPL4-1 | 1759 | 558 | YES | 1-16 | 8.635 | polysaccharide lyase family protein 4 | ADU33332.1 | *Dendroctonus ponderosae* | 723 | 0.0 | 64 |
| EscrPL4-2 | 1782 | 554 | YES | 1-23 | 463.296 | polysaccharide lyase family protein 4 | ADU33329.1 | *Dendroctonus ponderosae* | 861 | 0.0 | 73 |
| EscrPL4-3 | 2113 | 554 | YES | 1-19 | 270.890 | polysaccharide lyase family protein 4 | ADU33331.1 | *Dendroctonus ponderosae* | 679 | 0.0 | 60 |
| EscrPL4-4 | 1900 | 554 | YES | 1-21 | 1023.109 | polysaccharide lyase family protein 4 | ADU33330.1 | *Dendroctonus ponderosae* | 78 | 0.0 | 78 |

**Supplementary table** **S5**. Sequences used in the consensus tree. The sequences were obtained from GenBank or Vector Base.

| **Organism** | **Gene ID** | **Organism** | **Gene ID** |
| --- | --- | --- | --- |
| **GH9** | | | |

| *Polistes dominula* | XP_015184471.1 | *Polistes canadensis* | XP_014601854.1 |
| --- | --- | --- | --- |
| *Bombus terrestris* | XP_003402778.1 | *Bombus impatiens* | XP_012248155.1 |
| *Apis dorsata* | XP_006610535.1 | *Dufourea novaeangliae* | KZC10616.1 |
| *Fopius arisanus* | XP_011296916.1 | *Acromyrmex echinatior* | EGI63652.1 |
| *Acromyrmex echinatior* | XP_011057333.1 | *Temnothorax curvispinosus* | XP_024881045.1 |
| *Timema cristinae* | AMH40395.1 | *Antipaluria urichi* | AOV94253.1 |
| *Nilaparvata lugens* | XP_022200004.1 | *Tribolium castaneum* | KYB27092.1 |
| *Tribolium castaneum* | XP_008194455.1 | *Tribolium castaneum* | XP_001810693.2 |
| *Agrilus planipennis* | XP_025831030.1 | *Anoplophora glabripennis* | XP_018568196.1 |
| *Aretaon asperrimus* | AMH40356.1 | *Sipyloidea sipylus* | AMH40388.1 |
| *Medauroidea extradentata* | AMH40370.1 | *Aretaon asperrimus* | AMH40357.1 |
| *Sipyloidea sipylus* | AMH40389.1 | *Medauroidea extradentata* | AMH40367.1 |
| *Ramulus artemis* | AMH40383.1 | *Peruphasma schultei* | AMH40377.1 |
| *Peruphasma schultei* | AMH40376.1 | *Extatosoma tiaratum* | AMH40362.1 |
| *Ramulus artemis* | AMH40382.1 | *Medauroidea extradentata* | AMH40369.1 |
| *Sipyloidea sipylus* | AMH40391.1 | *Aretaon asperrimus* | AMH40361.1 |
| *Timema cristinae* | AMH40392.1 | *Antipaluria urichi* | AOV94250.1 |
| *Extatosoma tiaratum* | AMH40364.1 | *Medauroidea extradentata* | AMH40368.1 |
| *Aretaon asperrimus* | AMH40358.1 | *Sipyloidea sipylus* | AMH40386.1 |
| *Ramulus artemis* | AMH40380.1 | *Sipyloidea sipylus* | AMH40390.1 |
| *Peruphasma schultei* | AMH40375.1 | *Timema cristinae* | AMH40393.1 |
| *Extatosoma tiaratum* | AMH40365.1 | *Medauroidea extradentata* | AMH40371.1 |
| *Sipyloidea sipylus* | AMH40387.1 | *Aretaon asperrimus* | AMH40359.1 |
| *Peruphasma schultei* | AMH40378.1 | *Medauroidea extradentata* | AMH40366.1 |
| *Ramulus artemis* | AMH40381.1 | *Sipyloidea sipylus* | AMH40385.1 |
| *Peruphasma schultei* | AMH40373.1 | *Timema cristinae* | AMH40396.1 |
| *Timema cristinae* | AMH40394.1 | *Extatosoma tiaratum* | AMH40363.1 |
| *Medauroidea extradentata* | AMH40372.1 | *Sipyloidea sipylus* | AMH40384.1 |
| *Peruphasma schultei* | AMH40374.1 | *Ramulus artemis* | AMH40379.1 |
| *Aretaon asperrimus* | AMH40360.1 | *Antipaluria urichi* | AOV94251.1 |
| *Antipaluria urichi* | AOV94255.1 | *Antipaluria urichi* | AOV94254.1 |
| *Antipaluria urichi* | AOV94256.1 | *Antipaluria urichi* | AOV94252.1 |
| *Syntermes grandis* | AGP76431.1 | *Syntermes grandis* | AGP76432.1 |
| *Rhynchotermes bulbinasus* | AGP76429.1 | *Rhynchotermes bulbinasus* | AGP76428.1 |
| *Rhynchotermes bulbinasus* | AGP76427.1 | *Syntermes grandis* | AGP76433.1 |
| *Nasutitermes takasagoensis* | AA33708.1 | *Nasutitermes walkeri* | BAA33709.1 |
| *Globitermes sulphureus* | AGP76406.1 | *Globitermes sulphureus* | AGP76407.1 |
| *Constrictotermes cavifrons* | AGP76402.1 | *Constrictotermes cavifrons* | AGP76403.1 |
| *Hospitalitermes bicolor* | AGP76408.1 | *Hospitalitermes bicolor* | AGP76409.1 |
| *Hospitalitermes bicolor* | AGP76410.1 | *Nasutitermes corniger* | AGP76417.1 |
| *Nasutitermes sp.* | AGP76418.1 | *Microcerotermes crassus* | AGP76411.1 |
| *Anoplotermes schwarzi* | AGP76400.1 | *Anoplotermes schwarzi* | AGP76401.1 |
| *Grigiotermes hageni* | AGP76405.1 | *Subulitermes baileyi* | AGP76430.1 |
| *Sinocapritermes mushae* | BAD12012.1 | *Pericapritermes nitobei* | AGP76422.1 |
| *Pericapritermes sp.* | AGP76424.1 | *Pericapritermes sp.* | AGP76425.1 |
| *Pericapritermes sp.* | AGP76426.1 | *Sinocapritermes mushae* | BAD12013.1 |
| *Sinocapritermes mushae* | BAD12014.1 | *Nasutitermes takasagoensis* | BAD12011.1 |
| *Odontotermes hainanensis* | AGP76420.1 | *Odontotermes hainanensis* | AGP76421.1 |
| *Odontotermes formosanus* | AGP76419.1 | *Odontotermes formosanus* | BAD12010.1 |
| *Odontotermes formosanus* | BAD12009.1 | *Odontotermes formosanus* | BAD12008.1 |
| *Odontotermes formosanus* | ADB82658.1 | *Microtermes pallidus* | AGP76413.1 |
| *Macrotermes carbonarius* | AGP76414.1 | *Macrotermes gilvus* | AGP76415.1 |
| *Macrotermes subhyalinus* | AGP76416.1 | *Macrotermes barneyi* | AFD33365.1 |
| *Sphaerotermes sphaerothorax* | AGP76434.1 | *Sphaerotermes sphaerothorax* | AGP76435.1 |
| *Sphaerotermes sphaerothorax* | AGP76436.1 | *Sphaerotermes sphaerothorax* | AGP76437.1 |
| *Coptotermes formosanus* | ADB12483.1 | *Coptotermes gestroi* | AGS32241.1 |
| *Coptotermes acinaciformis* | AAK12339.1 | *Reticulitermes flavipes* | AAU20853.2 |
| *Reticulitermes speratus* | BAA31326.1 | *Reticulitermes speratus* | BAA34050.1 |
| *Coptotermes formosanus* | BAB40693.1 | *Coptotermes formosanus* | BAB40695.1 |
| *Coptotermes formosanus* | ACI45756.1 | *Coptotermes formosanus* | BAB40696.1 |
| *Microtermes pakistanicus* | AKV16371.1 | *Hodotermopsis sjostedti* | BAD11951.1 |
| *Hodotermopsis sjostedti* | BAD12003.1 | *Hodotermopsis sjostedti* | BAD12002.1 |
| *Mastotermes darwiniensis* | CAD54726.1 | *Mastotermes darwiniensis* | CAD54727.1 |
| *Mastotermes darwiniensis* | CAD54728.1 | *Mastotermes darwiniensis* | CAD54729.1 |
| *Mastotermes darwiniensis* | CAD54730.1 | *Neotermes koshunensis* | BAD12006.1 |
| *Neotermes koshunensis* | BAD12007.1 | *Salganea esakii* | BAG70027.1 |
| *Panesthia cribrata* | AAF80584.1 | *Panesthia angustipennis* | BAG70029.1 |
| *Panesthia cribrata* | AAF80585.1 | *Panesthia angustipennis* | BAG70030.1 |
| *Salganea esakii* | BAG70026.1 | *Teleogryllus emma* | ABV32557.1 |
| **GH28** | | | |
| *Ramulus artemis* | ANJ43643.1 | *Medauroidea extradentata* | ANJ43616.1 |
| *Ramulus artemis* | ANJ43644.1 | *Extatosoma tiaratum* | ANJ43595.1 |
| *Sipyloidea sipylus* | ANJ43661.1 | *Sipyloidea sipylus* | ANJ43660.1 |
| *Extatosoma tiaratum* | ANJ43594.1 | *Sipyloidea sipylus* | ANJ43659.1 |
| *Peruphasma schultei* | ANJ43631.1 | *Aretaon asperrimus* | ANJ43574.1 |
| *Ramulus artemis* | ANJ43639.1 | *Medauroidea extradentata* | ANJ43609.1 |
| *Extatosoma tiaratum* | ANJ43588.1 | *Sipyloidea sipylus* | ANJ43656.1 |
| *Peruphasma schultei* | ANJ43629.1 | *Peruphasma schultei* | ANJ43628.1 |
| *Sipyloidea sipylus* | ANJ43655.1 | *Sipyloidea sipylus* | ANJ43654.1 |
| *Ramulus artemis* | ANJ43638.1 | *Medauroidea extradentata* | ANJ43608.1 |
| *Extatosoma tiaratum* | ANJ43587.1 | *Extatosoma tiaratum* | ANJ43586.1 |
| *Sipyloidea sipylus* | ANJ43653.1 | *Ramulus artemis* | ANJ43637.1 |
| *Medauroidea extradentata* | ANJ43606.1 | *Medauroidea extradentata* | ANJ43605.1 |
| *Extatosoma tiaratum* | ANJ43584.1 | *Peruphasma schultei* | ANJ43627.1 |
| *Medauroidea extradentata* | ANJ43607.1 | *Extatosoma tiaratum* | ANJ43585.1 |
| *Extatosoma tiaratum* | ANJ43589.1 | *Extatosoma tiaratum* | ANJ43590.1 |
| *Medauroidea extradentata* | ANJ43610.1 | *Ramulus artemis* | ANJ43640.1 |
| *Aretaon asperrimus* | ANJ43572.1 | *Ramulus artemis* | ANJ43634.1 |
| *Medauroidea extradentata* | ANJ43603.1 | *Aretaon asperrimus* | ANJ43570.1 |
| *Extatosoma tiaratum* | ANJ43583.1 | *Sipyloidea sipylus* | ANJ43652.1 |
| *Peruphasma schultei* | ANJ43626.1 | *Ramulus artemis* | ANJ43635.1 |
| *Ramulus artemis* | ANJ43636.1 | *Aretaon asperrimus* | ANJ43571.1 |
| *Ramulus artemis* | ANJ43641.1 | *Medauroidea extradentata* | ANJ43611.1 |
| *Extatosoma tiaratum* | ANJ43591.1 | *Sipyloidea sipylus* | ANJ43657.1 |
| *Sipyloidea sipylus* | ANJ43658.1 | *Extatosoma tiaratum* | ANJ43592.1 |
| *Aretaon asperrimus* | ANJ43573.1 | *Peruphasma schultei* | ANJ43630.1 |
| *Ramulus artemis* | ANJ43642.1 | *Medauroidea extradentata* | ANJ43612.1 |
| *Medauroidea extradentata* | ANJ43615.1 | *Ramulus artemis* | ANJ43646.1 |
| *Medauroidea extradentata* | ANJ43617.1 | *Medauroidea extradentata* | ANJ43614.1 |
| *Medauroidea extradentata* | ANJ43613.1 | *Extatosoma tiaratum* | ANJ43593.1 |
| *Ramulus artemis* | ANJ43645.1 | *Extatosoma tiaratum* | ANJ43598.1 |
| *Extatosoma tiaratum* | ANJ43597.1 | *Extatosoma tiaratum* | ANJ43596.1 |
| *Sipyloidea sipylus* | ANJ43662.1 | *Peruphasma schultei* | ANJ43626.1 |
| *Ramulus artemis* | ANJ43635.1 | *Ramulus artemis* | ANJ43636.1 |
| *Aretaon asperrimus* | ANJ43571.1 | *Ramulus artemis* | ANJ43641.1 |
| *Medauroidea extradentata* | ANJ43611.1 | *Extatosoma tiaratum* | ANJ43591.1 |
| *Sipyloidea sipylus* | ANJ43657.1 | *Sipyloidea sipylus* | ANJ43658.1 |
| *Extatosoma tiaratum* | ANJ43592.1 | *Aretaon asperrimus* | ANJ43573.1 |
| *Peruphasma schultei* | ANJ43630.1 | *Ramulus artemis* | ANJ43642.1 |
| *Medauroidea extradentata* | ANJ43612.1 | *Medauroidea extradentata* | ANJ43614.1 |
| *Medauroidea extradentata* | ANJ43613.1 | *Medauroidea extradentata* | ANJ43615.1 |
| *Extatosoma tiaratum* | ANJ43593.1 | *Ramulus artemis* | ANJ43646.1 |
| *Medauroidea extradentata* | ANJ43617.1 | *Extatosoma tiaratum* | ANJ43598.1 |
| *Extatosoma tiaratum* | ANJ43597.1 | *Extatosoma tiaratum* | ANJ43596.1 |
| *Sipyloidea sipylus* | ANJ43662.1 | *Peruphasma schultei* | ANJ43633.1 |
| *Peruphasma schultei* | ANJ43632.1 | *Aretaon asperrimus* | ANJ43575.1 |
| *Ramulus artemis* | ANJ43650.1 | *Ramulus artemis* | ANJ43649.1 |
| *Ramulus artemis]\* | ANJ43651.1 | *Medauroidea extradentata* | ANJ43625.1 |
| *Aretaon asperrimus* | ANJ43582.1 | *Extatosoma tiaratum* | ANJ43602.1 |
| *Aretaon asperrimus* | ANJ43581.1 | *Extatosoma tiaratum* | ANJ43601.1 |
| *Aretaon asperrimus* | ANJ43578.1 | *Aretaon asperrimus* | ANJ43580.1 |
| *Aretaon asperrimus* | ANJ43579.1 | *Ramulus artemis* | ANJ43647.1 |
| *Medauroidea extradentata* | ANJ43618.1 | *Aretaon asperrimus* | ANJ43576.1 |
| *Medauroidea extradentata* | ANJ43620.1 | *Medauroidea extradentata* | ANJ43621.1 |
| *Aretaon asperrimus* | ANJ43577.1 | *Ramulus artemis* | ANJ43648.1 |
| *Medauroidea extradentata* | ANJ43622.1 | *Extatosoma tiaratum* | ANJ43599.1 |
| *Medauroidea extradentata* | ANJ43624.1 | *Extatosoma tiaratum* | ANJ43600.1 |
| *Medauroidea extradentata* | ANJ43623.1 | *Callosobruchus maculatus* | ADU33264.1 |
| *Callosobruchus maculatus* | ADU33265.1 | *Callosobruchus maculatus* | ADU33267.1 |
| *Callosobruchus maculatus* | ADU33266.1 | *Callosobruchus maculatus* | ADU33269.1 |
| *Callosobruchus maculatus* | ADU33268.1 | *Diabrotica virgifera virgifera* | XP_028140296.1 |
| *Diabrotica virgifera virgifera* | AHJ09936.1 | *Chrysomela tremula* | ADU33282.2 |
| *Chrysomela tremula* | ADU33279.2 | *Phaedon cochleariae* | CCJ09446.1 |
| *Gastrophysa viridula* | ADU33342.1 | *Chrysomela tremula* | ADU33278.1 |
| *Phaedon cochleariae* | CCJ09448.1 | *Phaedon cochleariae* | CCJ09447.1 |
| *Chrysomela tremula* | ADU33277.1 | *Chrysomela tremula* | ADU33276.1 |
| *Gastrophysa viridula* | ADU33344.1 | *Gastrophysa viridula* | ADU33343.2 |
| *Diabrotica virgifera virgifera* | XP_028131535.1 | *Diabrotica virgifera virgifera* | AHJ09933.1 |
| *Diabrotica virgifera virgifera* | AHJ09937.1 | *Diabrotica virgifera virgifera* | AHJ09927.1 |
| *Phaedon cochleariae* | CCJ09445.1 | *Phaedon cochleariae* | CCJ09449.1 |
| *Chrysomela tremula* | ACP18831.2 | *Gastrophysa viridula* | ADU33339.2 |
| *Leptinotarsa decemlineata* | AEX93414.1 | *Phaedon cochleariae* | CCJ09441.1 |
| *Phaedon cochleariae* | O97400.1 | *Gastrophysa viridula* | ADU33338.2 |
| *Chrysomela tremula* | ADU33280.2 | *Leptinotarsa decemlineata* | XP_023022574.1 |
| *Phaedon cochleariae* | CCJ09442.1 | *Chrysomela tremula* | ADU33281.1 |
| *Gastrophysa viridula* | ADU33340.1 | *Leptinotarsa decemlineata* | XP_023016332.1 |
| *Diabrotica virgifera virgifera* | AHJ09935.1 | *Diabrotica virgifera virgifera* | AHJ09928.1 |
| *Phaedon cochleariae* | CCJ09444.1 | *Gastrophysa viridula* | ADU33341.1 |
| *Chrysomela tremula* | ADU33275.1 | *Leptinotarsa decemlineata* | ADU33357.1 |
| *Diabrotica virgifera virgifera* | AHJ09929.1 | *Leptinotarsa decemlineata* | AHJ09926.1 |
| *Leptinotarsa decemlineata* | ADU33360.2 | *Leptinotarsa decemlineata* | ADU33362.1 |
| *Leptinotarsa decemlineata* | XP_023016647.1 | *Leptinotarsa decemlineata* | ADU33359.2 |
| *Leptinotarsa decemlineata* | ADU33356.1 | *Leptinotarsa decemlineata* | XP_023016648.1 |
| *Phaedon cochleariae* | CCJ09443.1 | *Leptinotarsa decemlineata* | ADU33363.2 |
| *Diabrotica virgifera virgifera* | AHJ09930.1 | *Sphenophorus levis* | AHC08666.1 |
| *Sphenophorus levis* | AHC08665.1 | *Sitophilus oryzae* | AAG35693.1 |
| *Sitophilus oryzae* | ADU33254.1 | *Dendroctonus ponderosae* | XP_019764756.1 |
| *Dendroctonus ponderosae* | ERL93974.1 | *Dendroctonus ponderosae* | XP_019764759.1 |
| *Dendroctonus ponderosae* | AEE61589.1 | *Rhynchophorus ferrugineus* | AMK48566.1 |
| *Dendroctonus ponderosae* | AEE63283.1 | *Sitophilus oryzae* | ADU33256.1 |
| *Sitophilus oryzae* | ADU33258.1 | *Sitophilus oryzae* | ADU33257.1 |
| *Sitophilus oryzae* | XP_030745830.1 | *Dendroctonus ponderosae* | XP_019757716.1 |
| *Dendroctonus ponderosae* | ADU33313.1 | *Dendroctonus ponderosae* | AEE61389.1 |
| *Dendroctonus ponderosae* | ENN78417.1 | *Dendroctonus ponderosae* | XP_019768950.1 |
| *Dendroctonus ponderosae* | ERL89408.1 | *Dendroctonus ponderosae* | XP_019756276.1 |
| *Dendroctonus ponderosae* | AEE61870.1 | *Dendroctonus ponderosae* | XP_019762278.1 |
| *Dendroctonus ponderosae* | XP_019753447.1 | *Dendroctonus ponderosae* | XP_019762770.1 |
| *Apriona japonica* | AHJ09940.1 | *Anoplophora glabripennis* | XP_018568795.1 |
| *Dendroctonus ponderosae* | XP_019761185.1 | *Dendroctonus ponderosae* | XP_019757385.1 |
| *Dendroctonus ponderosae* | ERL91880.1 | *Anoplophora glabripennis* | XP_018568799.1 |
| *Anoplophora glabripennis* | XP_018568797.1 | *Anoplophora glabripennis* | XP_018568798.1 |
| *Apriona japonica* | AHJ09939.1 | *Anoplophora glabripennis* | XP_018568805.1 |
| *Anoplophora glabripennis* | XP_018568806.1 | *Anoplophora glabripennis* | XP_018576819.1 |
| *Anoplophora glabripennis* | XP_018576136.1 | *Anoplophora glabripennis* | XP_023310577.1 |
| *Anoplophora glabripennis* | XP_018565019.1 | *Anoplophora glabripennis* | XP_018564981.1 |
| *Anoplophora glabripennis* | XP_018564895.1 | *Anoplophora glabripennis* | XP_018562870.1 |
| *Anoplophora glabripennis* | XP_018577959.1 | *Anoplophora glabripennis* | XP_018562871.1 |
| *Anoplophora glabripennis* | XP_018562875.1 | *Anoplophora glabripennis* | XP_018562894.1 |
| *Lygus lineolaris* | ABD63921.1 | *Lygus lineolaris* | ABD63920.1 |
| *Lygus lineolaris* | ABD63922.1 |  |  |
| **GH45** | | | |
| *Apriona japonica* | AHI15752.1 | *Batocera horsfieldi]* | AKH90729.1 |
| *Apriona germari* | AAN78326.1 | *Anoplophora chinensis* | AFN89565.1 |
| *Anoplophora glabripennis* | XP_018561275.1 | *Psacothea hilaris* | ALE71518.1 |
| *Apriona germari* | AAR22385.1 | *Apriona japonica* | AHI15753.1 |
| *Anoplophora glabripennis* | XP_018561265.1 | *Oncideres albomarginata chamela* | ADI24132.1 |
| *Mesosa myops* | AMA76413.1 | *Sitophilus oryzae* | XP_030747083.1 |
| *Sitophilus oryzae* | XP_030751361.1 | *Dendroctonus ponderosae* | XP_019754618.1 |
| *Dendroctonus ponderosae* | XP_019754620.1 | *Rhynchophorus ferrugineus* | ANU06045.1 |
| *Phaedon cochleariae* | CCJ09450.1 | *Leptinotarsa decemlineata* | ADU33350.1 |
| *Diabrotica virgifera virgifera* | AFI56547.1 | *Chrysomela tremula* | ADU33285.1 |
| *Phaedon cochleariae* | CCJ09451.1 | *Phaedon cochleariae* | CCJ09456.1 |
| *Leptinotarsa decemlineata* | ADU33345.1 | *Dendroctonus ponderosae* | XP_019766961.1 |
| *Dendroctonus ponderosae* | AEE61893.1 | *Rhynchophorus ferrugineus* | AMK48569.1 |
| *Phaedon cochleariae* | CCJ09453.1 | *Phaedon cochleariae* | CCJ09454.1 |
| *Phaedon cochleariae* | CCJ09452.1 | *Leptinotarsa decemlineata* | ADU33346.1 |
| *Dendroctonus ponderosae* | XP_019772608.1 | *Dendroctonus ponderosae* | AEE61846.1 |
| *Dendroctonus ponderosae* | XP_019771466.1 | *Dendroctonus ponderosae* | XP_019771468.1 |
| *Leptinotarsa decemlineata* | ADU33348.1 | *Leptinotarsa decemlineata* | XP_023029515.1 |
| *Leptinotarsa decemlineata* | ADU33347.1 | *Leptinotarsa decemlineata* | XP_023029506.1 |
| *Sitophilus oryzae* | XP_030751155.1 | *Sitophilus oryzae* | ADU33249.1 |
| *Sitophilus oryzae* | XP_030763222.1 | *Dendroctonus ponderosae* | XP_019757792.1 |
| *Dendroctonus ponderosae* | AEE61695.1 | *Phaedon cochleariae* | CCJ09455.1 |
| *Chrysomela tremula* | ADU33286.1 | *Gastrophysa viridula* | AZH23718.1 |
| *Gastrophysa viridula* | ADU33334.1 |  |  |
| **GH48** | | | |
| *Thermoclostridium stercorarium* | CAA93280.1 | *Caldicellulosiruptor saccharolyticus* | P22534.2 |
| *Hungateiclostridium thermocellum* | AAA23226.1  WP_020457708.1 | *Clostridium cellulovorans* | AAC38571.3 |
| *Ruminiclostridium cellulolyticum* | P37698.2  WP_015924275.1 | *Piromyces sp.* | AXH21508.1 |
| *Piromyces sp.* | AAN76734.1 | *Piromyces equi* | AAN76735.1 |
| *Neocallimastix patriciarum* | AEX92722.1 | *Neocallimastix sp.* | AXH21509.1 |
| *Pecoramyces ruminatium* | ANS13826.1 | *Streptomyces sp.* | WP_026243345.1 |
| *Streptomyces sp.* | WP_030897217.1 | *Gastrophysa viridula* | ADU33336.1 |
| *Gastrophysa atrocyanea* | BAE94320.1 | *Chrysomela tremula* | ADU33283.1 |
| *Gastrophysa viridula* | ADU33337.1 | *Leptinotarsa decemlineata* | ADU33353.1 |
| *Gastrophysa viridula* | ADU33335.1 | *Gastrophysa atrocyanea* | BAE94321.1 |
| *Chrysomela tremula* | ADU33284.1 | *Leptinotarsa decemlineata* | ADU33352.1 |
| *Diabrotica virgifera virgifera* | XP_028128323.1 | *Sitophilus oryzae* | ADU33252.1 |
| *Sitophilus oryzae* | XP_030746517.1 | *Dendroctonus ponderosae* | XP_019757754.1 |
| *Dendroctonus ponderosae* | XP_019757837.1 | *Dendroctonus ponderosae* | XP_019761706.1 |
| *Otiorhynchus sulcatus* | CAH25542.1 | *Leptinotarsa decemlineata* | ADU33354.1 |
| *Diabrotica virgifera virgifera* | XP_028152111.1 | *Dendroctonus ponderosae* | XP_019764094.1 |
| *Rhynchophorus ferrugineus* | AMK48558.1 | *Dendroctonus ponderosae* | XP_019756174.1 |
| *Rhynchophorus ferrugineus* | AMK48560.1 | *Rhynchophorus ferrugineus* | AMK48585.1 |
| *Dendroctonus ponderosae* | ERL93248.1 | *Caldicellulosiruptor saccharolyticus* | AAA91086.1 |
| *Anoplophora glabripennis* | XP_023310951.1 |  |  |
| **CE8** | | | |
| *Klebsiella aerogenes* | CCG31089.1 | *Klebsiella variicola* | ADC59471.1 |
| *Klebsiella michiganensis* | AFN31494.1 | *Escherichia coli* | AAC73859.1 |
| *Escherichia coli* | CBG33614.1 | *Kosakonia sacchari* | AHJ76577.1 |
| *Cronobacter turicensis* | CBA29343.1 | *Dickeya dadantii* | ADM98093.1 |
| *Dickeya dadantii* | AHJ74673.1 | *Pectobacterium atrosepticum* | AIK12029.1 |
| *Serratia sp.* | ESN64780.1 | *Actinoplanes sp.* | AGZ41791.1 |
| *Dendroctonus ponderosae* | XP_019757645.1 | *Dendroctonus ponderosae* | XP_019757646.1 |
| *Dendroctonus ponderosae* | AEE62955.1 | *Sphenophorus levis* | AHC08664.1 |
| *Rhynchophorus ferrugineus* | AMK48559.1 | *Sitophilus oryzae* | XP_030748053.1 |
| *Dendroctonus ponderosae* | XP_019761741.1 | *Sphenophorus levis* | AHC08664.1 |
| *Rhynchophorus ferrugineus* | AMK48559.1 | *Sitophilus oryzae* | XP_030748053.1 |
| *Dendroctonus ponderosae* | XP_019761741.1 | *Dendroctonus ponderosae* | AEE62090.1 |
| *Sitophilus oryzae* | ADU33260.1 | *Rhynchophorus ferrugineus* | AMK48575.1 |
| *Sitophilus oryzae* | ADU33261.1 | *Dendroctonus ponderosae* | AEE62813.1 |
| *Dendroctonus ponderosae* | ERL95168.1 | *Sitophilus oryzae* | XP_030762872.1 |
| *Sitophilus oryzae* | ADU33263.1 | *Ralstonia solanacearum* | AAA25984.1 |
| *Xanthomonas oryzae* | AAW75950.1 | *Actinoplanes friuliensis* | AGZ41791.1 |
| *Actinoplanes missouriensis* | BAL87114.1 | *Nocardiopsis dassonvillei* | ADH66823.1 |
| *Amycolatopsis mediterranei* | AEK43000.1 | *Actinoplanes sp.* | AEV84054.1 |
| *Bacillus infantis* | AGX04064.1 | *Bacillus infantis* | AGX04063.1 |
| *Bacillus paralicheniformis* | AGN37876.1 | *Asticcacaulis excentricus* | ADU15052.1 |
| *Hirschia baltica* | ACT60297.1 | *Saccharophagus degradans* | ABD80206.1 |
| *Teredinibacter turnerae* | ACR11378.1 | *Cellvibrio japonicus* | ACE84027.1 |
| *Caulobacter segnis* | ADG09008.1 | *Dickeya dadantii* | ADM98093.1 |
| *Pectobacterium atrosepticum* | AIK15043.1 | *Kosakonia sacchari* | AHJ76577.1 |
| *Kosakonia sacchari* | AHJ74673.1 | *Marinomonas mediterranea* | ADZ92341.1 |
| *Marinomonas posidonica* | AEF53511.1 | *Alteromonas macleodii* | AFS37793.1 |
| *Aspergillus niger* | CAK47976.1 | *Aspergillus niger* | CAK38387.1 |
| *Aspergillus niger* | CAK44519.1 | *Penicillium rubens* | CAP83067.1 |
| *Penicillium rubens* | CAP86666.1 | *Aspergillus nidulans* | EAA60938.1 |
| *Aspergillus nidulans* | EAA63358.1 | *Botrytis cinerea* | CCD34306.1 |
| *Botrytis cinerea* | CCD50540.1 | *Botrytis cinerea* | CCD51623.1 |
| *Thermothelomyces thermophila* | AEO60762.1 | *Thermothelomyces thermophila* | AEO61202.1 |
| *Melanopsichium pennsylvanicum* | CDI54281.1 | *Moniliophthora perniciosa* | AFO55205.1 |
| *Thielavia terrestris* | AEO66258.1 | *Thielavia terrestris* | AEO70721.1 |
| *Bipolaris zeicola* | AAD43340.1 | *Fusarium fujikuroi* | CCT65443.1 |
| *Podospora anserina* | CAP66743.1 | *Fusarium fujikuroi* | CCT75603.1 |
| *Fusarium fujikuroi* | CCT65443.1 | *Sporisorium reilianum* | CBQ73920.1 |
| *Lichtheimia ramosa* | CDS04799.1 | *Enterobacter hormaechei* | KHM55643.1 |
